# Supplementary material for: The implicit power of positive thinking: The effect of positive episodic simulation on implicit future expectancies
Source: PLoS One. 2024 Apr 30;19(4):e0298817. doi: 10.1371/journal.pone.0298817 (PMC11060537; doi:10.1371/journal.pone.0298817)
Supplement: Attachment — Submitted filename: Review Comments.docx [file pone.0298817.s001.docx]

Review Summary:

This article examines the short-term impact of positive episodic simulation training on implicit optimism in a community sample of college students. It aims to address an important gap in the existing literature on episodic specificity inductions and future thinking training, which have primarily relied on explicit measures to index training/induction effects. There are important methodological, design, and analysis issues in the present study that should be addressed and clarified. In addition, there are a number of assertions that require additional context or support, without which some conclusions appear overstated or unwarranted based on the present findings. I have outlined specific comments by section below:

Introduction

1. The authors discuss the use of episodic simulation inductions and future specificity training to modify future expectancies, but do not sufficiently clarify the time scale of these effects throughout the Introduction (pages 3-5). Given these effects are notoriously short-lived, this is important context to provide for the current study and general avenue of research.
   - Please provide greater clarity about the time scale of these effects in the Introduction , and address the brief time scale as a limitation in the Discussion section (both for the current work and such procedures in general).
2. The authors rightly identify a limitation of self-report measures for future expectancies, but do not fully clarify how implicit measures like the IRAP or FT-IRAP overcome demand characteristics or reflect automatic beliefs (page 6):
   - The authors should make clear in the introduction and/or methods 1) how automatic beliefs are reflected by the FT-IRAP, 2) how that relates to speed or accuracy of responding, 3) and the relevance of the consistent vs. inconsistent trials to the indexing of automatic/optimistic orientations. For instance, if someone is implicitly optimistic about a future event, how is that reflected in their responding on a consistent or inconsistent trial for different trial types?
   - The authors should also elaborate how the measures used here are not subject to demand characteristics; for example, clarifying how directing participants to respond in either a consistent or inconsistent manner (and forcing them to respond correctly) is not imposing a demand characteristic.
3. I would encourage adding some explanation to the Introduction and/or Methods justifying using a neutral imagery condition for a control for the PST group (rather than a negative imagery condition for example). In particular, the authors should address why they feel the neutral imagery condition is an appropriate control for PST, given that a neutral vs positive comparison does not allow for dissociating influences of valence from arousal.
4. The final statement in the Introduction (page 7: “If positive episodic simulation is to form a useful tool... its beneficial effect…should not vary as a function of depressive symptom level”) seems at odds with the literature. The authors should justify their reasoning for predicting that training effects should benefit everyone regardless of depression symptom level. In particular, they should address the possibility that if people are not “sufficiently” depressed they may not see much benefit of positive simulation training (because they may already be at ceiling). This could be returned to in the Discussion section with regard to the positivity bias ceiling effect observed in this study at T1.

Methods

1. More information is needed about the demographic makeup of the sample and whether demographic characteristics were consistent across groups. Sex/gender composition and race/ethnicity should be reported for the overall sample and by group if that information is available. If it is not available, that should be addressed in the paper.
   - The authors need to address whether the Imagery groups were matched for age, sex/gender or race/ethnicity, and depressive symptom level (and provide results of supporting statistical tests). If the groups were not matched for any of these variables, the authors should account for these potential confounds in the analyses if possible.
2. More information is needed about depressive symptom characteristics of the sample and imagery groups. If there are clinical cutoffs or symptom severity ranges for the CESD-R scale those should be provided, and the symptom constellation for each group (and overall sample) should be clearly delineated. Whether the groups were matched for symptom severity should be addressed; and any differences should be accounted for in the analyses.
   - If individuals with clinically concerning levels of depressive symptomatology were not represented in the current sample, the authors should consider whether a certain degree of symptomatology is necessary to avoid a potential baseline ceiling effect in optimistic orientation; and similarly whether a certain level is necessary in order for participants to see any benefit of the training. This should be addressed in the Discussion section.
3. The authors should address whether clinical depression history was assessed and explain why/why not. This is particularly important to address considering the role of depressive symptomatology was a secondary aim.
4. Given that participants were shown the FT-IRAP stimuli until they responded (and were asked to correct their responses before the trial would advance), and that the breaks between FT-IRAP blocks were self-paced, it seems there is potential for substantial variation in timing between participants. The authors should address what efforts were taken to control for timing differences in their analyses.
   - This should be addressed both in terms of variation in exposure time to different trial types on the FT-IRAP, *and* for variation in the amount of time passed between the start of the FT-IRAP and Imagery tasks.
   - For example, were the self-paced rest breaks timed on the FT-IRAP ? How did the authors account for potential effects of time/passage of time differing across subjects or different iterations?
   - Similarly, since participants were shown FT-IRAP stimuli until a response was made (and were asked to correct their responses before the trial would advance), total exposure time to different stimuli and trial types may have varied between participants. How was this addressed?
5. Both the FT-IRAP and the Imagery tasks use a small stimulus set in a highly repeated fashion, creating the potential for habituation, practice effects, and interference from previous exposures or conflicting response demands. The authors should provide justification for these design decisions, as well as the decision to repeat stimuli in each of the different FT-IRAP trial types (where some have conflicting response demands). The impact of such design choices on the results and whether they represent potential confounds or limitations should be addressed in the Discussion section.
6. Please elaborate on how the VAMS were scored and how the VAMS change-scores were calculated.
7. Please address whether a baseline VAMS was obtained before or after the first FT-IRAP, to assess for potential influence of mood on FT-IRAP (and vice versa). If there was not a baseline VAMS, please address in the Discussion section whether any influence of current mood on FT-IRAP performance could be expected, or if the FT-IRAP might have any mood induction effects of its own.
8. Please describe your correction for multiple comparisons and state any associated thresholds.
9. Please clearly state how the optimistic bias was operationalized, and explain what the difference between consistent and inconsistent trial types reflects.

Results

1. The authors assert that disparities in baseline biases between groups made it necessary to examine DIRAP change scores between successive time points rather than raw data (p. 13). However, there are **no statistically significant disparities in baseline biases** reported between groups for any trial type. The only potential bias difference I see reported is actually not significant with appropriate correction for multiple comparisons. As written, these results do not indicate evidence of baseline disparities in bias, and call into question the use of DIRAP change scores for all subsequent analyses.
2. The authors mention the CESD-R was transformed to correct skew (p. 13), but no skewness/kurtosis statistics have been provided. Please report skewness/kurtosis statistics showing skew before and after transformation, to demonstrate that the transformation was necessary and appropriately improved skew.
3. As currently written, it’s unclear why the authors used Time 2 vs Time 3 to operationalize the “sustained” PST effect in the “Maintenance of PST effect over a short delay” section (p. 14). The authors should consider repeating this analysis using Time 1 as the baseline comparison against Time 3; or if comparing successive timepoints is still desired, a difference of differences approach is another alternative (i.e., Time 2-Time 1 vs Time 3-Time 1). Please ensure that any changes to this analysis are mirrored in the “Changes in Mood” analyses (p. 14-15). Otherwise, please provide sufficient justification for the decision to use Time 2 vs Time 3 to operationalize the sustained PST effect.
4. The authors should clearly state their rationale for including CESD-R as a covariate in the analyses; it’s unclear from the reported results whether there is sufficient statistical justification. Was there an interaction of CESD-R with group, or a correlation of CESD-R with one of the variables of interest? At the very least testing a group difference in CESD-R would seem important to demonstrate that both groups were statistically equivalent in terms of depression symptom levels.
5. Please report the behavioral means for each group at each time point for each of the main measures (VAMS, DIRAP values; vividness and emotionality ratings for the imagery tasks; RTs for the different FT-IRAP trial types if available). Consider adding a table.
6. Given the secondary aim focused on depressive symptom severity, please indicate if there was any correlation of CESD-R with optimistic orientation (either at baseline or after the imagery tasks), or with performance on any of the FT-IRAP trial types more generally. Similarly, was CESD-R correlated with emotionality or vividness on either of the imagery tasks?
7. Were there vividness and emotionality ratings at multiple time points across the Imagery tasks? If so, why were the time points not included in the analysis?

Discussion

1. Please comment on potential limitations of using such few stimuli in such a highly repeated manner in both the FT-IRAP and imagery procedures (in terms of habituation, practice effects, potential interference effects of previous exposures, etc) and how this may have impacted engagement with the tasks or influenced key outcome variables.
   - Specifically, the authors should address whether repeated exposure to stimuli in all trial types on the FT-IRAP (consistent/inconsistent, expect/don’t expect) had the potential to introduce confounds in the primary DIRAP measures by requiring participants to overcome previous response demands or associations. Did this repeated exposure in conflicting task conditions have the potential to introduce interference, either from previous emotional responses or behavioral responses? And what impact could that interference (or overcoming such interference) have had on the main DIRAP measures used to index optimistic orientation (DIRAP values and change scores)?
   - The authors should also address how repetition of stimuli within the Imagery conditions may have influenced engagement with the training or mood induction effects.
2. Given that participants were shown the FT-IRAP stimuli until they responded (and made a correct response), and that the breaks between FT-IRAP blocks were self-paced, it seems there is potential for substantial variation in timing between participants. The authors should address what efforts were taken to control for timing differences in their analyses; and what limitations such confounds may pose for the study conclusions. If timing variation was not controlled or accounted for in the analyses, this should be addressed and noted as a limitation. This should be addressed both in terms of variation in exposure time to different trial types on the FT-IRAP, *and* for variation in the amount of time passed between the start of the FT-IRAP and Imagery tasks.
3. Please discuss limitations about obtaining vividness and emotionality ratings at the end of each imagery procedure (rather than trial by trial), and any limitations from using the VAMS as a measure of mood. Comment on what degree of fluctuations you expected to capture in mood by operationalizing it as positive or negative valence (rather than continuous scores). Discuss whether an initial baseline mood assessment would have been helpful for addressing potential confounding influence of mood with the FT-IRAP (and vice versa).
4. The jigsaw task seems to have been intended as a filler task to provide a washout period from the PST. However, images of famous places may be rewarding/positively valenced. Please comment on any confounding influence or limitations posed by the use of positively valenced images in for the jigsaw task (rather than neutral images).

Figures

1. No figure captions have been provided.
2. Please indicate significant effects.

Word Choice & Typos

1. The phrase “faulty prospections” appears in a few places throughout the manuscript (pages 4, 18). I would urge the authors to consider changing this phrase instead to “biased prospections” or something to that effect. I’m concerned the original phrasing implies something is inherently wrong or flawed about such prospections, or that people with depression are “faulty” by extension; which I’m sure was not the authors’ intention. Indeed in some cases, a negative prospection bias may be accurately/appropriately informed by someone’s past experiences or current situation (such as life hardships); in which case it would not be considered faulty but rather an accurate assessment.
2. On page 3 (first paragraph): In the sentence, “Research has shown that future expectancies can be enhanced by engage…”; change engage to *engaging*.
